# Pediatric Upper Cervical Spine Trauma: A 10-Year Retrospective Review at a Pediatric Trauma Center

**DOI:** 10.7759/cureus.20995

**Published:** 2022-01-06

**Authors:** Sazid Hasan, Muhammad Waheed, Ameen K Suhrawardy, Collin Braithwaite, Lamia Ahmed, Philip Zakko, Jad G Khalil, Ehab S Saleh

**Affiliations:** 1 Department of Orthopedic Surgery, Oakland University William Beaumont School of Medicine, Rochester, USA; 2 Public Health, Wayne State University School of Medicine, Detroit, USA; 3 Department of Orthopedic Surgery, Beaumont Hospital, Royal Oak, USA

**Keywords:** treatment, injury mechanism, retrospective study, pediatrics, trauma, cervical spine

## Abstract

Background

Traumatic upper cervical spine injuries (tUCSI) are generally caused by high-impact injuries to the C1-C2 vertebral level. The current literature is limited with regards to comparing epidemiological trends, treatment options, and overall outcomes for tUCSI within the pediatric cohort. The purpose of this study was to analyze pediatric tUCSI epidemiological data, potential variations in treatment and patient outcomes, and to evaluate any specific trends that may be clinically relevant.

Methodology

We conducted a retrospective cohort study on pediatric patients ages 1 day to 16 years old, admitted for tUCSI over the past 10 years (1/2011 to 1/2021) at a Midwest level 1 trauma center. Retrospective data was queried using ICD-9 and ICD-10 diagnosis codes for tUCSI. Children were stratified into three age groups: Group 1 - Infants and Toddlers (children under three years of age); Group 2 - Young Children (children between three and seven years of age); Group 3 - Juveniles and Adolescents (children between the ages of seven and 16). Numerical data and categorical variables were summarized and the normality of the distribution of data was evaluated using the Anderson-Darling normality test. Differences between the age groups were examined using either an unpaired, independent Two-Sample t-test or Unpaired Mann-Whitney U test. Pearson’s chi-squared or Fisher’s exact tests were used to compare categorical data between groups.

Results

Forty total patients were included in the final analysis, 23 female (57.5%) and 17 male (42.5%). The mean age was 11 ± 4 (range 2-16). Overall, the most common mechanism of injury was a motor vehicle collision (n=16, 40%), followed by sports injury (n=13, 32.5%), falls (n=6, 15%), and unknown mechanism (n=5, 12.5%). The most common mechanism of injury in young children was a fall (n=4, 57.5%,* p*<0.001). Adolescents and Juveniles significantly suffer from sports injuries compared to young children (n=13, 39.4%, *p*=0.043). Mechanisms of injuries presented with unique associated concomitant injuries. The most common associated sites of injuries were lower cervical spine (n=31, 77.5%), and skull injury (n=4, 10%). The vast majority of these cases were managed nonoperatively (pain medication and non-operative cervical orthosis) (n=36, 90%). Mortality and morbidity rates from tUCSI were rare in our cohort (n=1, 2.5%).

Conclusion

This study found that the majority of pediatric tUCSI patients can be managed nonoperatively, with dislocations and spinal instability being the most common indications for operative management. Commonly used non-operative external fixation methods include cervical collars and Minerva jackets. Our cohort showed very low mortality and morbidity rates, however, these preliminary results will require validation by future prospective multicenter studies.

## Introduction

Upper cervical spine injuries in pediatric patients, defined as injuries to the C1-C2 vertebral level, are typically associated with high morbidity and mortality rates. The mortality rate of cervical spine trauma in pediatric patients ranges from 16% to 18%, with a higher rate in pediatric patients sustaining upper cervical spine injuries [1]. Pediatric patients are more susceptible to cervical trauma than adults with up to 80% of pediatric vertebral injuries occurring within the cervical spine, while cervical spine trauma in adults only accounts for 30% to 40% of spine injuries [1,2].

These trends can be attributed to the anatomical differences between pediatric and adult patients. In pediatric patients, the anatomy of the cervical spine changes continuously with their growth. Pediatric spines are characterized by increased elasticity of the interspinous ligaments, posterior joint capsule, and cartilaginous endplates [2,3]. Specifically, wedge-shaped vertebral bodies and the horizontal orientation of the facet joints may increase upper cervical spine trauma in children [2]. In addition, pediatric patients have a relatively larger head mass, resulting in a higher center of gravity and a greater propensity for high cervical spine injury rates following dynamic movements or sport [3]. Thus, this unique anatomy of the pediatric vertebral column poses a relatively increased risk for upper cervical spine injuries when compared to the adult spine [4,5].

Infantile pediatric patients, particularly those under 36 months, have been shown to be a distinct population, with studies showing differences in location of the injury, gender distribution, and treatment recommendations [6,7]. On the other hand, the cervical spine acquires its adult characteristics between the ages of eight to 10 years with these children presenting with injury patterns similar to those seen in adults [6,8,9]. Younger pediatric patients are mostly injured in car or motorcycle crashes, whereas older patients suffer significantly more injuries during sports [10].

Radiologic evaluation of the pediatric spine can be challenging due to the wide range of normal anatomic variants and synchondroses, along with various biomechanical forces that are unique to children [11]. Neurologic examination is critical in the timely and accurate diagnosis of a spinal cord injury that, although uncommon, can be devastating, especially if a diagnostic delay allows the injury to worsen [3]. Supported by the high rate of absence of radiologic abnormalities in children with a cervical spine injury, any child whose mental status does not allow accurate cervical spine evaluation must be assumed to already have a cervical spine injury until proven otherwise by objective clinical evaluation [3].

Conservative/non-operative treatment, consisting of nonsteroidal anti-inflammatory medications, pain medication, cervical thoracic orthosis (Minerva jacket), cervical collar braces (Miami J, Aspen), Halter’s traction, and outpatient follow-up have been shown to play an important role in traumatic upper cervical spine injuries (tUCSI) management. Nevertheless, the possibility of needing surgical treatment still exists in rare cases [12]. Common surgical procedures undertaken for pediatric tUCSI include the application of halo tractions for vertebral reduction and realignment, anterior or posterior fusion, and vertebral decompression [1].

Unfortunately, the literature sparsely touches on the epidemiology, injury associations, treatment modalities, and outcomes for pediatric tUCSI. Thus, the purpose of this study was to evaluate tUCSI in our institution’s pediatric population over a 10-year period. Because tUCSI within the pediatric population is marked by a low incident rate, future multicenter prospective studies are needed to improve our overall knowledge of this subject.

## Materials and methods

We conducted a retrospective cohort study on pediatric patients ages 1 day to 16 years old, admitted for tUCSI over the past 10 years (1/2011 to 1/2021) at a level 1 trauma center in midwestern United States. Electronic medical records (EMR) were retrieved and reviewed from a single institution. Retrospective data was queried from EPIC, an electronic clinical patient record system, using ICD-9 and ICD-10 diagnosis codes for atlantoaxial instability, atlantoaxial rotatory displacement, odontoid fracture, atlas fracture, and transverse ligament injury, traumatic spondylolisthesis of axis (Hangman fracture), occipital condyle fracture, and occipital cervical dislocations. Children aged 1 day to 16 years with tUCSI diagnosed as one of the aforementioned ICD-10 codes and who were admitted as inpatients were included in this study. All EMRs were reviewed for emergency room notes, orthopedic clinical notes, telemedicine notes, imaging documents, and reports. An independent review of each patient’s chart was performed to gather demographic information such as patient age, gender, and race as well as clinical parameters including vitals upon admission, mechanism of injury, associated injuries, level of injury, imaging modalities utilized, type of intervention, associated complications, discharge diagnosis, length of stay, and overall patient outcomes upon discharge. The results were then stratified into three age groups for statistical analysis: Group 1 - Infants and Toddlers (children under three years of age); Group 2 - Young Children (children between three and seven years of age); Group 3 - Juveniles and Adolescents (children between the ages of seven and 16). This study was approved by the institutional review board: approval number 2021-185.

Numerical data were summarized as mean ± standard deviation and categorical variables were summarized as n (%). The normality of the distribution of data was evaluated using the Anderson-Darling normality test. Differences between the age groups were examined using an unpaired, independent Two-Sample t-test for the continuous outcomes with normal distributions while an Unpaired Mann-Whitney U test was used for continuous outcomes with non-normal distributions. Pearson’s chi-squared or Fisher’s exact tests were used to compare categorical data between groups. Statistical significance was set at p < 0.05. Analyses were performed using Minitab v. 21.1.0 software (Minitab Inc, State College, Pennsylvania) and Microsoft Excel v. 2103 (Microsoft Corporation, Redmond, Washington).

## Results

A total of 40 patients were included in our final analysis of which 23 (57.5%) were female and 17 (42.5%) were male (Table 1). The mean age at admission was 11 ± 4 ranging between 3 to 16. No patient was under three years of age (Infant and Toddler group), seven (17.5%) were between the ages of three years and seven years, and 33 (82.5%) were aged over seven years. There were 26 white patients (65%), 10 black patients (25%), one Asian patient (2.5%), and three unspecified patients (7.5%). The mean length of stay was 3.28 days ± 8.74 which ranged from a minimum of 0 days to a maximum of 54 days. The majority of patients stayed for one day (n=26, 65%).

**Table 1:** Demographic and clinical data of 40 children who suffered tUCSI. The young children group were children aged between three and seven years; the juvenile and adolescent group were between seven and 16 years. * Two-sample Pearson’s chi-squared test or Fisher's Exact Test + Unpaired Two-Sample t-test È Unpaired Wilcoxon Mann–Whitney U test SD: Standard Deviation; MVA: Motor vehicle accidents.

	Total Group (n=40)	Young Children Group (n=7)	Juvenile and Adolescent Group (n=33)	Comparison between young children group and juvenile and adolescent group (p-value)
Female, n (%)	23 (57.5)	2 (28.6)	21 (63.6)	0.088*
Mean Age, years (SD)	11 (4)	3.42 (0.98)	12.97 (2.79)	<0.001^+^
Race				
White, n (%)	26 (65)	3 (42.8)	23 (69.7)	0.176*
Black, n (%)	10 (25)	1 (14.3)	9 (27.3)	0.471*
Asian, n (%)	1 (2.5)	1 (14.3)	0 (0)	0.175*
Unspecified, n (%)	3 (7.5)	2 (28.6)	1 (3.03)	0.0737*
Mean Length of Stay, days (SD)	3.28 (8.74)	3.71 (5.50)	3.19 (9.37)	0.846^È^
Injury Mechanism				
MVA, n (%)	16 (40)	2 (28.6)	14 (42.4)	0.497*
Sport, n (%)	13 (32.5)	0 (0)	13 (39.4)	0.043*
Fall, n (%)	6 (15)	4 (57.14)	2 (6.06)	0.001*
Idiopathic, n (%)	5 (12.5)	1 (14.3)	4 (12.1)	0.875*
ICD Diagnosis				
Ligamental Sprain, n (%)	34 (82.5)	5 (71.43)	29 (72.5)	0.268*
Spinal instabilities, n (%)	3 (7.5)	0 (0)	3 (9.1)	1*
Closed dislocation, n (%)	1 (2.5)	1 (14.3)	0 (0)	0.552*
Dislocation of cervical spine, n (%)	1 (2.5)	0 (0)	1 (3.03)	1*
Dislocation of cervical vertebrae, n (%)	1 (2.5)	1 (14.3)	0 (0)	0.552*
Imaging				
X-ray, n (%)	21 (52.5)	3 (42.8)	18 (54.5)	0.574*
CT, n (%)	12 (30)	2 (28.6)	10 (30.3)	0.928*
MRI, n (%)	12 (30)	2 (28.6)	4 (12.1)	0.268*
Treatment				
Pain Medication, n (%)	29 (72.5)	5 (71.43)	24 (72.7)	0.944*
Cervical Orthoses, n (%)	10 (25)	2 (28.6)	8 (24.2)	0.81*
Surgery, n (%)	4 (10)	1 (14.3)	3 (9.1)	0.552*
Follow-Up Recommendation, n (%)	27 (67.5)	4 (57.14)	23 (69.7)	0.519*
Patient Outcomes				
Discharged Stable, n (%)	39 (97.5)	6 (85.7)	33 (100)	0.175*
Death, n (%)	1 (2.5)	1 (14.3)	0 (0)	0.175*
Complications, n (%)	2 (5)	2 (28.6)	0 (0)	0.027*

At the time of admission, children presented with an average blood pressure of 116.3 ± 12.75 mmHg systolic over 68.5 ± 11.96 diastolic with young children presenting with an elevated average systolic blood pressure of 122.3 mmHg compared to juvenile and adolescents at 115 mmHg (p>0.05). Young children similarly presented with an elevated mean pulse (91 beats per minute) and respiratory rate (25.43 breaths per minute) compared to juveniles and adolescents (83.3 beats per minute; 22.76 breaths per minute) (p=0.277; p=0.272).

**Figure 1:**
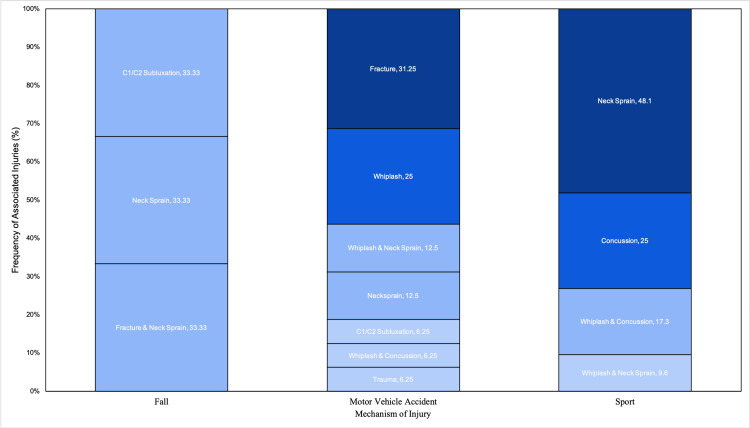
Mechanism of upper cervical spine trauma and associated concomitant injuries.

The most common mechanism of injury overall was motor vehicle collision (n=16, 40%), followed by sports injury (n=13, 32.5%), a fall (n=6, 15%), and an unknown mechanism (n=5, 12.5%) (Table 1). The most common mechanism of injury in young children was a fall (n=4, 57.5%, p<0.001), while sports injuries and motor vehicle accidents (MVA) accounted for the most common mechanism in juveniles and adolescents (42.4%). Children who experienced falls commonly presented with torticollis (n=3, 50%). Juvenile and adolescents were also far more prone to sports injuries (13, 39.4%) than young children (0, 0%) (p=0.043). Mechanisms of injuries presented with different associated injuries and frequencies (Figure 1). Falls were most commonly associated with C1/C2 subluxation, isolated neck sprains, and fractures with neck sprains (33.33% each), MVA with fractures and whiplash (31.25%, 25% respectively), and Sports with neck sprain and concussion (46.15%, 23.08% respectively). 

**Figure 2:**
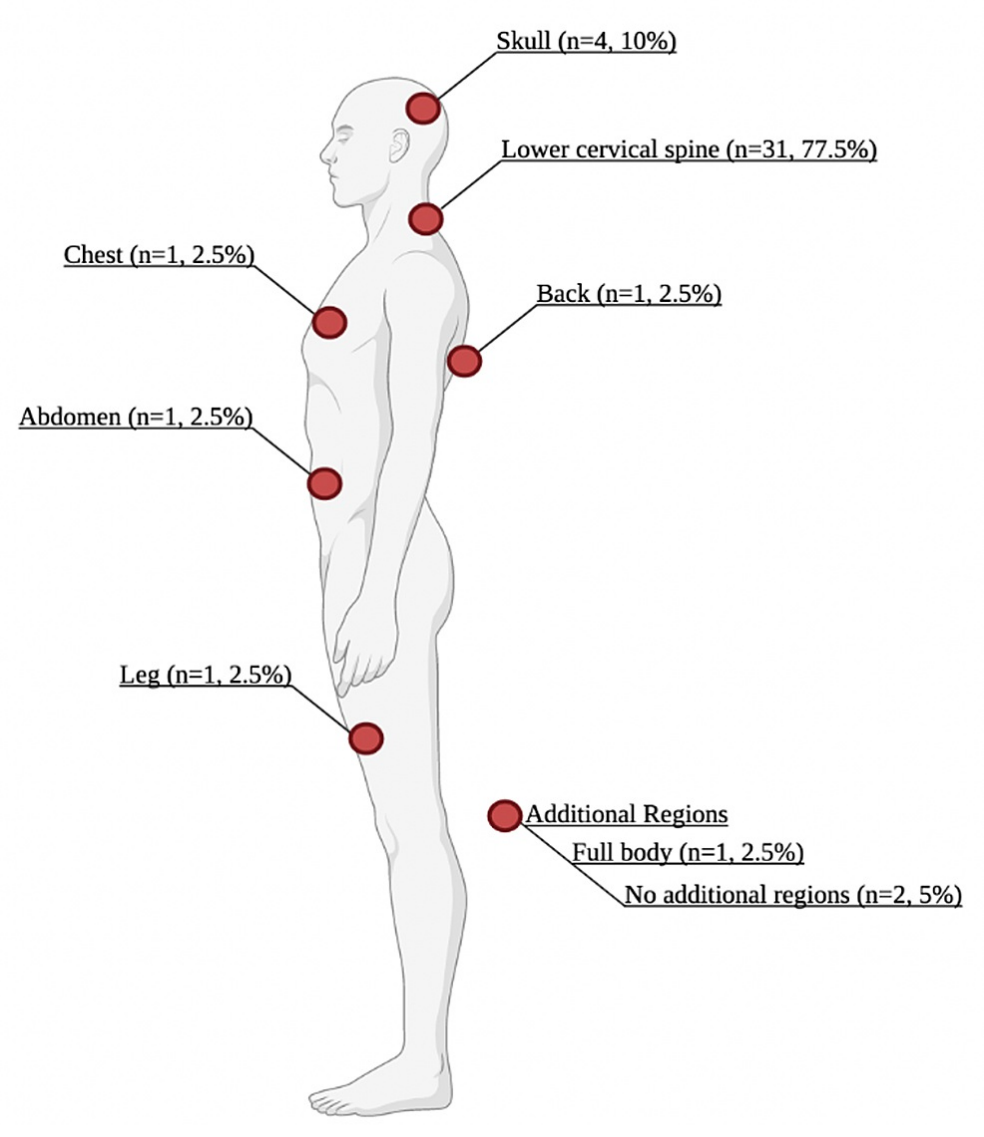
Associated sites of injury following traumatic upper cervical spine injury. Created with BioRender.com

There were 34 (85%) patients with ligament sprains, three (7.5%) with spinal instabilities, and three (7.5%) with vertebral dislocations with no significant differences between groups. Patients would frequently have other locations of injury in addition to the C1/C2 site including the lower cervical spine (n=31, 77.5%), skull (n=4, 10%) as well as back, leg, chest, abdomen, and full body (n=1, 2.5%) (Figure 2). Only two patients presented with no additional sites of injury. Various imaging modalities were used with X-ray (n=21, 52.5%) being the most frequent, followed by both CT (n=12, 30%) and MRI (n=12, 30%). The most common treatments provided were pain medications (n=29, 72.5%), followed by conservative cervical orthosis (n=10, 25%). In our study, four patients (10%) were operated on for cervical spinal instability, ligamental sprain, and dislocation. Chi-square analysis revealed that surgery was more common in those that had a dislocation or spinal instability, versus those that had a sprain of ligaments (p<0.001). Twenty-seven patients (67.5%) were told to follow up with their primary care physician (PCP). Most patients were discharged in stable condition (n=39, 97.5%) with one patient dying from multiple, major complications associated with a motor vehicle accident that resulted in multiple injuries including subarachnoid hemorrhages, atlantoaxial and atlantooccipital dislocation, left-sided pneumothorax, bilateral pulmonary contusions, shocked bowel, left femoral fracture, bilateral obturator ring fractures, distal left clavicle fracture, and bilateral first rib fracture.

## Discussion

This retrospective study elucidates many underlying principles behind tUCSI in pediatric patients including mechanisms of injury, associated concomitant injuries, additional cervical spine sites of injury, as well as approach to treatment. The overall incidence of pediatric cervical spine trauma is about 1.5% of all traumatic injuries in children [3,13]. The leading cause of traumatic pediatric cervical spine injuries in the literature is blunt trauma (95%), primarily due to motor vehicle collisions (61%) [14]. Our data also confirmed that trauma resulting from motor vehicle collisions is the overall most common mechanism of injury. The second most common cause of pediatric cervical spine injury differs depending on age. Falls are the second most common cause of upper cervical spine injury in children under eight years of age (18% for < 8 years of age vs 11% > 8 years), while sports injuries are the second leading cause in older children (3% for < 8 years of age vs 20% > 8 years) [3,14]. Similarly, our study parallels these etiologies in the young children and the juvenile/adolescent group, with falls representing 57.1% of injuries in the young children group and sports representing 39.4% of injuries in the juvenile/adolescent group. This pattern follows logical reasoning for the respective prevalence of mechanism of injuries in these two groups. Young children may have gaits not yet fully developed and a higher center of gravity with an increased relative head to body ratio, predisposing them to falls with greater mechanical impact on the cervical spine. For adolescents, engagement in sports is a common cause of spinal trauma. In the patients with sports-related injuries, we found trauma resulting from contact sports with high momentum impact such as hockey, football, wrestling, and volleyball, as well as from performative sports with dynamic movements and high angular velocity such as dance, gymnastics, cheerleading, and trampoline.

Almost all patients in the study had accompanying musculoskeletal injuries concurrent with their upper cervical spine trauma, with only two out of 40 patients having no other additional injury. The mechanisms of injury correlated differently with the associated concomitant injuries, as seen in Figure 1. For falls, torticollis was found in 50% of all injuries, meaning that young children who underwent upper cervical spine injury had an increased risk of acquiring torticollis. This is thought to be caused by inflammation that leads to laxity of the cervical ligaments and capsular structures at the atlantoaxial level and permits rotational deformity in young children [15]. For sport-related cervical trauma, we found neck sprains or concussions in over 90% of patients with upper cervical spine injury, likely a consequence of the impact that the musculoskeletal system absorbs in the high-velocity movements of sports. Finally, in MVA injuries, there was a high association with fractures and whiplash injuries as a result of the high-energy collision. Overall, lower cervical spine injuries (77.5%) and skull injuries (10%) were the most common additional injuries in the study population as seen in Figure 2.

Despite the occurrence of concomitant injuries, patient outcomes were largely positive. Thirty-nine of 40 patients were discharged in stable condition, with the majority (67.5%) of patients discharged within a day or less after admission. Only two patients, both in the young children group, had subsequent complications from the upper cervical spine trauma. Unfortunately, one death occurred due to the traumatic injury in a young child who was struck by a vehicle while walking, representing a mortality rate of 2.5%. In comparison, the literature indicates that mortality rates of pediatric upper cervical spine trauma patients range from 16% to 18% [4,16]. New pediatric trauma protocols implemented by our center in the past 10 years might be a factor in our cohort’s lower mortality rate. Our institution uses the new expert consensus cervical spine protocol as outlined by the Pediatric Spine Expert Group [17,18]. Any pediatric patient presenting with a Glasgow coma scale score less than 15 will be provided a cervical collar and undergo an X-ray and stat MRI. CT is not frequently performed to reduce radiation exposure in the pediatric population. Any patient that is cleared with a cervical collar is recommended for a follow-up in two weeks.

The management and treatment of pediatric cervical spine trauma are notably more complex when compared to the adult population. Prior studies, such as Reilly et al. (published 2007) had commented on the lack of well-established protocols with regards to management and treatment approaches [17]. The distinctive cervical anatomy, mechanisms of injury, as well as inconclusive imaging modalities, create an ongoing challenge to establish agreed-upon protocols for pediatric cervical spine trauma patients [17-19]. However, a great collaboration by a panel of fellowship-trained physicians, the Pediatric Cervical Spine Clearance Working Group published a report in 2019 which provides guidance in institutional cervical spine clearance protocols [18]. The guide takes into account that the management of traumatic pediatric upper cervical spine injuries is guided based on a variety of factors, including but not limited to the type and mechanism of injury, age of patient, clinical assessment (including neurological status, Glasgow coma scale), and imaging findings [18,19].

Conservative approaches play a central role in the treatment and management of pediatric cervical spine injuries. In an 18-year, two-center retrospective study, Tomaszewski et al. (n=6) found that nonoperative management using conservative cervical orthosis resulted in satisfactory subjective and functional outcomes with no observed complications [20]. Conservative management was largely effective for the study population in this review. Thirty-six patients (90%) were treated nonoperatively, which is remarkable considering 39 (97.5%) of patients were discharged in a stable condition. Our data analysis shows that most pediatric upper cervical spine injuries can be managed with conservative measures (n=36, 90%) and a minority required open surgical procedures (n=4, 10%). We also demonstrated that most pediatric patients with upper cervical spine trauma could be discharged in a stable condition with cervical orthoses, nonsteroidal anti-inflammatory medications, pain medications, and outpatient follow-up with orthopedic surgery or neurology.

The immediate focus of management after an injury is to immobilize the cervical spine to reduce cervical instability and prevent subsequent progression of any neurological deficits. When evaluating cervical spine trauma, certain patterns of injury may allow for nonoperative management. Commonly used external fixation methods include the Halo orthosis devices, Halter’s traction, and Minerva body jackets [21,22]. A study by Odent et al. found that patients with odontoid fractures who were treated with a halo device (12 patients) had a better overall recovery and experienced fewer complications compared to those who were managed operatively (three patients) [23]. Many other studies have found similar results, highlighting the principle that external fixation remains the preferred modality of treatment for pediatric odontoid injuries [24-26]. Of the 10 patients in our study who were stabilized with cervical orthoses, Minerva vest, and Miami J collar braces were utilized.

In cases of severe cervical spine fractures or dislocations, unstable injuries, or progressive deformity, surgical interventions via internal fixation or surgical decompression are warranted as definitive treatment [14,19,25]. The innate pediatric cervical anatomy may pose a greater challenge for pin or screw placement when compared to the adult spine [26]. However, the advent of technological advancements in preoperative and intraoperative imaging has assisted clinicians in discerning the anatomical variations in each patient and ultimately guiding operative management [18,26]. Operative management in pediatric upper cervical spine patients differs based on the level of injury. Of the four patients who were operated on in our study, two patients underwent halo vest placement, one patient underwent halo traction placement. The one child who underwent halo traction placement presented with C1 rotatory subluxation relative to C2 with post-traumatic changes and sclerosis. A lateral mass of their C1 was perched on the anterior aspect of C2 and their C1 vertebral articular facet for dens was laterally displaced relative to the odontoid process. The patient was placed in halo traction for two weeks until closed reduction was achieved. They were then placed in a halo vest for 26 days until discharged stable. One patient underwent transoral odontoidectomy for decompression of the brainstem and occipital to C4 fusion to achieve spinal stability after presenting with upward migration of dens, basilar invagination, and cervical stenosis.

Another important consideration for clinicians who encounter pediatric trauma is that concomitant injuries should be assessed for when evaluating pediatric patients with upper cervical spine trauma, particularly the lower cervical spine and skull injuries. Diagnosing and treating concurrent lower cervical spine trauma may be especially critical in preventing more serious consequences of spinal cord damage. Evaluation for concomitant injuries should be considered with the mechanism of the cervical spine injury, as fracture and whiplash are associated with MVA, neck sprain and concussion are associated with sports injury. In addition, it is important to consider those complications are more likely to arise in young children who experience upper cervical spine injury.

There are a few limitations to this study. This is a retrospective review that covers one center over the span of 10 years, suggesting the demographics, injury characteristics, treatment protocols, and outcomes may be unique to the geographic region covered by this medical center. In addition, the small sample size can contribute to statistical limitations and reduced generalizability to the overall pediatric population. Although short-term follow-up did show an overall good outcome, this study did not maintain long-term follow-up with patients to monitor progression or improvement of cervical injury. Nevertheless, the trends delineated in this study are paralleled in other studies in the pediatric cervical spine literature and warrant more investigation into long-term sequelae of pediatric upper cervical injury.

## Conclusions

In our retrospective cohort study on 40 pediatric patients with traumatic upper cervical spine injury, we found that the vast majority of patients were managed with nonoperative treatment and discharged in stable condition often within one day of admission. Surgical intervention was pursued in four patients, and complications were only reported in two patients who were in the young children group (3 to 7 years old). Concomitant injuries were found in 95% of patients, with associated injuries correlating differently with the mechanism of cervical trauma. The lower cervical spine and the skull were the most common sites of additional injury in children with upper cervical spine trauma. Our cohort showed very low mortality and morbidity rates, however, these results require further corroboration by future prospective multicenter studies.
